# Ultrahigh sensitivity and layer-dependent sensing performance of phosphorene-based gas sensors

**DOI:** 10.1038/ncomms9632

**Published:** 2015-10-21

**Authors:** Shumao Cui, Haihui Pu, Spencer A. Wells, Zhenhai Wen, Shun Mao, Jingbo Chang, Mark C. Hersam, Junhong Chen

**Affiliations:** 1 Department of Mechanical Engineering, University of Wisconsin–Milwaukee, 3200 N Cramer Street, Milwaukee, Wisconsin 53211, USA; 2 Department of Materials Science and Engineering, Northwestern University, Evanston, Illinois 60208, USA; 3 Department of Chemistry, Northwestern University, Evanston, Illinois 60208, USA

## Abstract

Two-dimensional (2D) layered materials have attracted significant attention for device applications because of their unique structures and outstanding properties. Here, a field-effect transistor (FET) sensor device is fabricated based on 2D phosphorene nanosheets (PNSs). The PNS sensor exhibits an ultrahigh sensitivity to NO_2_ in dry air and the sensitivity is dependent on its thickness. A maximum response is observed for 4.8-nm-thick PNS, with a sensitivity up to 190% at 20 parts per billion (p.p.b.) at room temperature. First-principles calculations combined with the statistical thermodynamics modelling predict that the adsorption density is ∼10^15^ cm^−2^ for the 4.8-nm-thick PNS when exposed to 20 p.p.b. NO_2_ at 300 K. Our sensitivity modelling further suggests that the dependence of sensitivity on the PNS thickness is dictated by the band gap for thinner sheets (<10 nm) and by the effective thickness on gas adsorption for thicker sheets (>10 nm).

## Abstract (web summary)

Phosphorene is one of a growing number of 2D materials with high potential for device applications. Here, the authors report a sensor composed of phosphorene nanosheets, showing a high sensitivity to NO_2_ in dry air and also demonstrate that the sensitivity depends on the nanosheet thickness.

## Graphical abstract

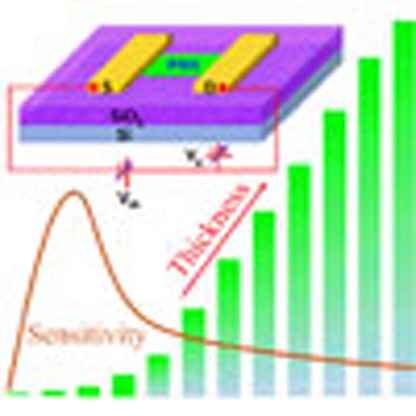

Two-dimensional (2D) nanosheets constructed by a single or several atomic layers, such as graphene and metal dichalcogenides (for example, MoS_2_), represent an emerging class of materials and have attracted significant attention because of their unique structures and physicochemical properties^1^,^2^,^3^,^4^, which are associated with large surface areas and abundant active sites. These properties endow 2D nanosheets with exciting prospects for a variety of applications in electronics, sensors, catalysis and energy storage/conversion devices^5^,^6^,^7^. Previous important findings have stimulated extensive interest among physicists, chemists and engineers to further explore novel 2D nanosheets such as nitrides^8^, oxides^9^, phosphates^10^ and carbides^11^.

Black phosphorus (BP), the most stable allotrope of phosphorus, is a material with individual atomic layers stacked together through van der Waals interactions. Bulk BP has a structure similar to graphite and is possibly the only elemental substance that can be mechanically exfoliated into an ultrathin nanosheet other than graphite. Bulk BP is a *p*-type semiconducting material with a direct band gap of ∼0.3 eV (refs 12, 13, 14) and the field-effect mobility of BP is dependent on its thickness^13^. Recent studies have shown the band gap of BP is tunable from 0.3 eV for bulk BP to 2.0 eV for phosphorene (monolayer BP), depending on the number of layers, suggesting property control in the 2D limit^12^,^13^. Few-layer phosphorene also has been demonstrated as an outstanding and reliable transistor material with a drain current modulation on the order of 10^5^ and a mobility up to 1,000 cm^2^ V^−1^ s^−1^, and it exhibits well-developed current saturation in the transfer characteristics at room temperature^13^. Therefore, phosphorene nanosheets, thanks to their thickness-dependent direct band gap and high on/off current ratio, may offer highly desirable properties and great potential for field-effect transistor (FET) and related applications, such as in a complementary metal–oxide–semiconductor inverter^14^.

Here we demonstrate the phosphorene nanosheet (PNS) FET devices using the ‘Scotch tape' method and report their potential applications in chemical sensing in a dry air environment. The PNS sensor showed surprisingly outstanding performance for detecting NO_2_ in dry air at room temperature at the parts per billion (p.p.b.) level. First-principles calculations and statistical thermodynamics modelling were performed to understand the underlying sensing mechanism for such superior PNS sensors. To probe the optimum thickness of PNS for the best sensing performance, we measured the sensing response of PNS with various thicknesses and an interesting trend was observed. Simulation was also performed to understand the thickness-dependent sensing responses.

## Results

### Structure of PNS and PNS FET chemical sensors

**Figure 1:**
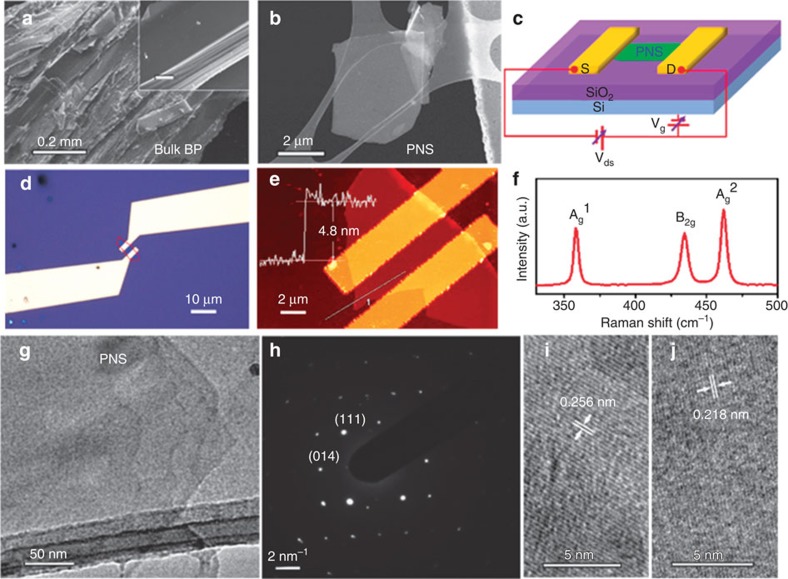
PNS FET sensor device fabrication and characterization. (**a**) Scanning electron microscopy (SEM) image of bulk BP. The inset is a magnified image showing the layered structure; scale bar, 10 μm. (**b**) SEM image of exfoliated PNS. (**c**) Schematic of an FET device based on the PNS and the circuit for electrical and sensing measurements. (**d**,**e**) Optical microscopy and AFM images of the PNS sensor device showing that the PNS electrically bridges the gold electrodes. The profile in **e** indicates the PNS has a thickness of 4.8 nm. (**f**) Raman spectrum of PNS. (**g**,**h**,**i,j**) TEM image, selected area electron diffraction (SAED) pattern and high-resolution TEM (HRTEM) images of PNS. The two HRTEM images (**i**,**j**) demonstrate two representative lattice spacings of PNS.

The PNS used for the sensor device was exfoliated from commercial bulk BP (Fig. 1a) through the ‘Scotch tape' method^15^. The FET was fabricated by depositing the PNS (Fig. 1b) onto a 300-nm-thick oxide layer on a Si substrate and then metal contacts were defined by the standard electron-beam lithography process. Figure 1c shows a schematic of the PNS FET device and the circuit for electrical and sensing measurements. The PNS bridges the gap of gold fingers, serving as the conductance channel (Fig. 1d). The measured thickness of the PNS by atomic force microscopy (AFM) is 4.8 nm (Fig. 1e), suggesting that our PNS sample is multilayer. The Raman scattering measurement on the PNS in Fig. 1f identifies three Raman peaks located at around 358, 434 and 462 cm^−1^, corresponding with the A_g_^1^, B_2g_ and A_g_^2^ modes of the pristine PNS, respectively, in good agreement with previous observations^14^,^16^. The structure of the exfoliated PNS was also characterized by scanning electron microscopy and transmission electron microscopy (TEM) on a holey carbon grid (Fig. 1b,g). The selected area electron diffraction pattern demonstrates the single crystalline structure of the PNS (Fig. 1h). Figure 1i,j show the high-resolution TEM images of PNSs with lattice spacings of 0.256 and 0.218 nm, corresponding with the (111) and (020) planes of side-centred orthorhombic BP, respectively^17^.

### The dynamic-sensing characteristics of PNS devices

Before the sensing measurements, the FET transfer curve of the 4.8-nm-thick PNS device was first measured in vacuum. We provided a fixed bias across the PNS conductive channel and swept the gate voltage from –60 to 60 V. The results demonstrate that the channel state switched from ‘on' to ‘off' state with a current drop on the order of 10^5^ (Supplementary Fig. 1a). The device was also measured to verify that the contacts between the PNS and metal electrodes were Ohmic (Supplementary Fig. 1b), because a Schottky barrier at the interface between the electrode and the 2D nanomaterial of the FET device suppresses charge transport^18^, while a low-resistance Ohmic contact provides a direct injection channel at the interface and thus minimizes the interface's influence on the intrinsic sensing properties of the 2D nanomaterial.

**Figure 2:**
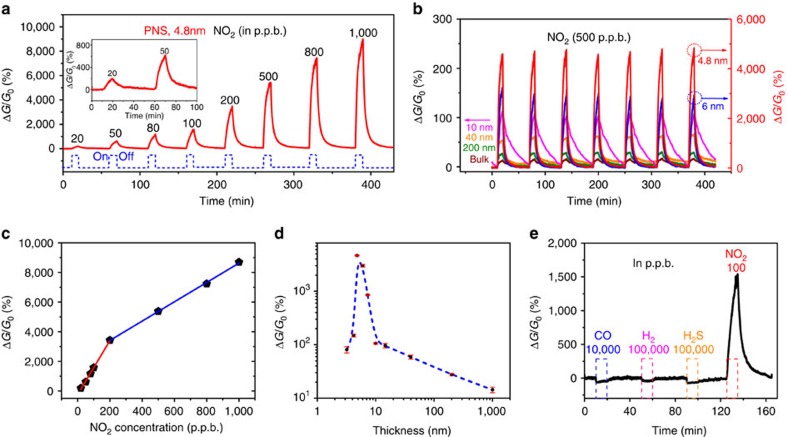
Sensing characteristics of PNS sensors. (**a**) Dynamic response curves of relative conductance change versus time for NO_2_ concentrations ranging from 20 to 1,000 p.p.b. (balanced in dry air) for PNS (4.8 nm). A drain-source voltage of 0.6 V was applied to the device. The dashed line demonstrates the ‘on/off' of NO_2_ gas. The sensitivity here is defined as the differential response between Δ*G*/*G*_0_=0 in the air environment at the first cycle and the Δ*G*/*G*_0_ at the end of gas ‘off' for each concentration. (**b**) Thickness-dependent multi-cycle responses of the PNS sensor to 500 p.p.b. NO_2_. (**c**) Calibration curves of the 4.8-nm-thick PNS sensors to NO_2_ gas. (**d**) Trend line for the thickness-dependent multi-cycle responses in **b**. (**e**) Dynamic-sensing response curve of the 4.8-nm PNS to various gases, including 10,000 p.p.b. CO, 100,000 p.p.b. H_2_, 10,000 p.p.b. H_2_S and 100 p.p.b. NO_2_. The sensor shows a much higher response to NO_2_ compared with other gases.

The dynamic-sensing response of the PNS sensor was investigated for detecting NO_2_ in dry air at room temperature. A constant source-drain bias of 0.6 V was applied to the sensor and the electrical conductance change of the device on exposure to NO_2_ was monitored and recorded as the sensing signal. In Fig. 2a, the dynamic-sensing response or relative conductance change (Δ*G*/*G*_0_) versus time for 4.8-nm-thick PNS is demonstrated, with the NO_2_ concentration varying from 20 to 1,000 p.p.b. (balanced in dry air). The electrical conductivity of the PNS sensor increased on exposure to NO_2_. As NO_2_ is an oxidizing gas, it withdraws electrons from the PNS when NO_2_ adsorbs on the PNS surface. As the PNS behaves as a *p*-type semiconductor at room temperature (Supplementary Fig. 1a) and is used as the conductance channel in our sensor device, the electron transfer from the PNS to NO_2_ effectively increases the charge carrier (hole) concentration in the PNS, thereby increasing its electrical conductivity. The PNS sensor response to NO_2_ is similar to those of other *p*-type semiconducting sensors, such as graphene-based materials^19^. The same measurements were also performed for 40-nm-thick and bulk BP sensors, and similar results were observed (Supplementary Fig. 2). We also plotted the overlapping response curves (Supplementary Fig. 3) using the same data in Fig. 2a. It clearly shows the response amplitude increases with increasing gas concentrations and the sensor can completely recover to its initial state in dry air.

Our 4.8-nm-thick PNS sensor has an excellent response of 190% to a concentration level as low as 20 p.p.b. and the sensitivity can reach 1,600% at a concentration of 100 p.p.b. The lower detection limit could be pushed down to an even lower level. The excellent sensing response is competitive when compared with most other high-performance NO_2_ gas sensors as summarized in Supplementary Table 1. For example, the lower detection limit of voltage-activated reduced graphene oxide (RGO) is 50 p.p.b. (ref. 19); the calculated lower detection limits of RGO conjugated with Cu_2_O nanowires and chemically modified graphene are 64 and 70 p.p.b., respectively^20^,^21^; the calculated lower detection limit of semiconduting single-walled carbon nanotubes to NO_2_ is 44 p.p.b. (ref. 22); and multiple In_2_O_3_ nanowires had a response to NO_2_ down to a low p.p.b. level (5 p.p.b.)^23^. Besides graphene-based sensors, 2D MoS_2_ nanosheets decorated with Pt nanoparticles also have been reported as a 2D material for room-temperature NO_2_ sensors with a calculated lower detection limit of 2 p.p.b. (ref. 24). It is noteworthy that for the multiple In_2_O_3_ nanowire sensors^23^ with a lower detection limit down to 5 p.p.b., the sensitivity at 20 p.p.b. was ∼45%, much lower than the senstivity of 190% for our 4.8-nm PNS sensor.

The band gap of PNS can be tuned by varying its thickness, which could significantly affect the sensitivity. To probe the trend of sensitivity versus the thickness of PNS and to observe the optimum thickness for sensing, we have measured the sensing performance for different PNS thicknesses ranging from a 3.2-nm-thick PNS sensor to bulk BP sensors against 500 p.p.b. NO_2_ (Fig. 2b and Supplementary Fig. 4). Interestingly, a higher sensitivity was observed with decreasing PNS thickness until 4.8 nm, after which the sensitivity dramatically decreased, which demonstrated that the sensitivity of PNS sensors was significantly dependent on its thickness. Figure 2d plots the trend line of the response dependence on the PNS thickness. We clearly observed that the sensitivity dramatically increased when the PNS was thinner than ∼10 nm and the response was maximized at around 5 nm. Then, a significant drop occurred for the even thinner PNS sensors. This could be explained by the influence of the tunable band gap and we will explore the mechanism using theoretical calculations in the later section.

Figure 2c plots the derived sensor response as a function of NO_2_ concentration for the 4.8-nm PNS. The sensing sensitivity increases with the increasing concentration of NO_2_ from 20 to 1,000 p.p.b. Interestingly, the sensitivities for concentrations from 20 to 200 p.p.b. and from 200 to 1,000 p.p.b. can fit well into two linear curves with different slopes, respectively. This similar phenomenon was also observed for the 40-nm-thick PNS and bulk BP sensor (Supplementary Fig. 5) except that the critical gas concentration is lowered to ∼100 p.p.b. The larger slope curve (high-sensitive mode) in the relatively low concentration range (≤200 p.p.b.) demonstrates a relatively higher increase rate in the sensitivity, whereas the sensitivity increases more slowly (low-sensitive mode) in the relatively high concentration range (⩾200 p.p.b.). As electrical conductance is the product of carrier concentration and carrier mobility, we hypothesize that the switch from the high-sensitive mode to the low-sensitive mode could be attributed to the degradation of carrier mobility due to the scattering effects of adsorbed NO_2_ molecules (Supplementary Fig. 6 and Supplementary Note 1). When PNS is exposed to a relatively low concentration of NO_2_, the degradation of carrier mobility could be negligible because of sparse NO_2_ molecule adsorption, corresponding to the high-sensitive mode. However, when the NO_2_ concentration is sufficiently high, the degradation of carrier mobility becomes so significant that it leads to a reduced rate of increase in sensitivity (low-sensitive mode) due to abundant NO_2_ molecule adsorption.

To demonstrate the strong specific adsorption to NO_2_, the PNS sensor was studied by measuring the same sensor with several other gases, including 10,000 p.p.b. CO, 100,000 p.p.b. H_2_ and 10,000 p.p.b. H_2_S. The sensing measurement was carried out in the same manner as that for measuring NO_2_ and the dynamic sensor responses to various gases are shown in Fig. 2e. Although the concentrations of CO, H_2_ and H_2_S are significantly higher than that of NO_2_ (100 p.p.b.), the sensor showed weak responses to all gases except NO_2_, demonstrating the strong specific adsorption ability of the PNS sensor to NO_2_.

The stability of PNS sensors in air is critical for practical applications. To probe the stability, we aged PNS in ambient air (relative humidity ∼42% at 22 °C) and dry air for a certain period of time, respectively. AFM was used to track the morphology change. For the PNS in ambient air, we observed many bubbles on the PNS surface within 17 h, which is consistent with an earlier report^25^, whereas we did not find an obvious change for PNS exposed to a dry airflow, even after 2.5 days (Supplementary Fig. 7). This indicates that water in air affects the PNS probably due to the relatively hydrophilic nature of the PNS surface. To further prove the stability of our sensor, we tested the 40-nm PNS sensor to 500 p.p.b. NO_2_ in a dry air condition with different aging durations (0 day, 2 days and 5 days). The dynamic sensing responses demonstrate that the sensitivities are similar, indicating a high stability of PNS sensors in dry air (Supplementary Fig. 8).

### Ultrahigh NO_2_ adsorption ability of phosphorene

**Figure 3:**
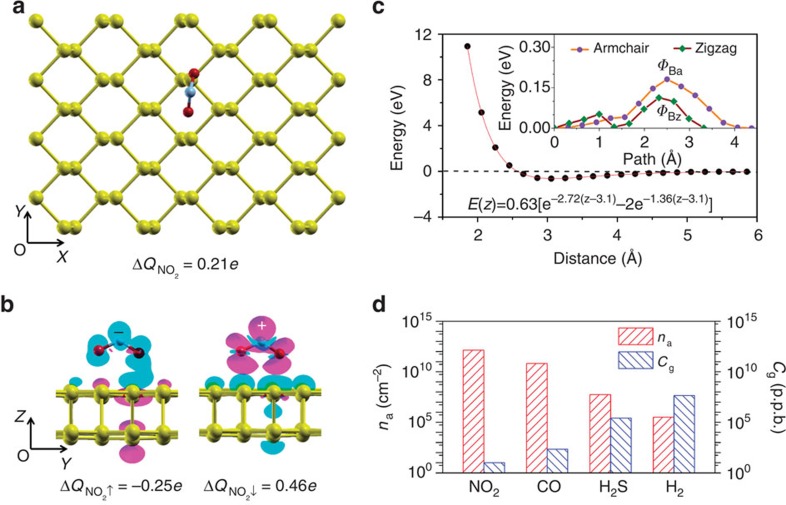
DFT calculations of NO_2_ adsorption on phosphorene surface. (**a**) Top view of the adsorption of a single NO_2_ molecule on the phosphorene surface with oxygen atoms pointing downwards. (**b**) Side views of difference charge density contour plot (1.25 × 10^−3^ electron per Bohr^3^) for the spin-up (left) and spin-down (right) states with the Mulliken charge transfer indicated. The violet and cyan colour-coded regions indicate the charge accumulation and depletion, respectively. The yellow, red and light blue balls represent the P, O and N atoms, respectively. (**c**) The Morse potential type of interaction strength between the single NO_2_ molecule and the phosphorene surface with respect to the distance (defined as the height difference between the N atom and the top P atoms), as shown in **a** with the fitted curve and parameters. The inset shows the total energy change as a function of the distance from the initial ground state, as the NO_2_ molecule migrates to its first nearest neighbouring ground state adsorption site with the energy barriers Φ_Ba_ and Φ_Bz_ of 0.18 and 0.08 eV along the armchair (*X* direction in **a**) and zigzag (*Y* direction in **a**) directions, respectively. (**d**) Left axis: the adsorption densities for the four target gas species (NO_2_, CO, H_2_S and H_2_) on phosphorene with the gas concentration fixed at 20 p.p.b. at 300 K, respectively; right axis: concentrations of CO, H_2_S and H_2_ required to reach the same adsorption density of NO_2_ (2.8 × 10^12^ cm^−2^) at 20 p.p.b.

To elucidate the underlying mechanism of the superior sensitivity of PNS towards NO_2_, density functional theory (DFT) calculations were performed on monolayer and multilayer phosphorene, to study the adsorption strength (the dependence of which on the layer number will be discussed in a later section). As the NO_2_ molecule is paramagnetic^26^,^27^,^28^, the spin-polarized effect is considered here; otherwise the binding energy will be overestimated due to the spin dipole moment interaction. Figure 3a presents the energetically favoured molecular configuration for NO_2_ adsorption. The DFT-calculated binding energy *E*_b_ is up to 0.63 eV per molecule (Supplementary Fig. 9 and Supplementary Note 2 for other less energetically favoured adsorption sites and molecular orientations), which is quite large compared with those on graphene (0.21 eV per molecule) or RGO (typically 0.11∼0.29 eV per molecule depending on the oxygen-containing functional groups)^29^. This can be understood from the atomic orbital bonding perspective (Supplementary Fig. 10 and Supplementary Note 3). The difference charge density in Fig. 3b shows that the *p*_z_ orbitals in phosphorus atoms surrounding the NO_2_ molecule accept (donate) electrons from (into) the NO_2_ molecule for the spin-up (spin-down) state on adsorption and the net Mulliken charge transfer is 0.21*e*.

It is noteworthy that the recent theoretical work by Kou *et al*.^26^ also suggested that phosphorene is superior for gas sensing. In their work, they calculated both the gas-binding energy and transport properties of monolayer phosphorene before and after the gas adsorption. Here we further correlate the binding energy quantitatively with the superior sensitivity by establishing a statistical thermodynamics model to evaluate the gas-adsorption density. For crystalline materials, the adsorbed gas molecule from its free phase is treated as the lattice gas and the interaction between the gas molecule and the solid surface (that is, the crystalline material) is described by the Morse potential


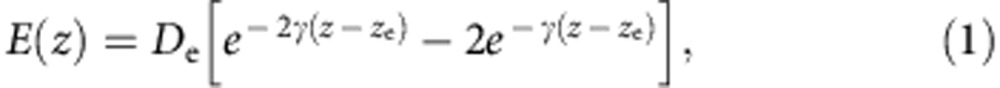


where *D*_e_ is the potential well depth, *γ* is the fitting parameter and *z*_e_ is the distance between the gas molecule and the solid surface in its equilibrium state (Fig. 3c). Gas molecules are considered to be adsorbed on the solid surface only when *E*(*z*) is negative and the eigenstates *E*_n,z_ should be identified by solving the Schrödinger equation. In the canonical ensemble, the gas adsorption density *n*_a_ is^30^


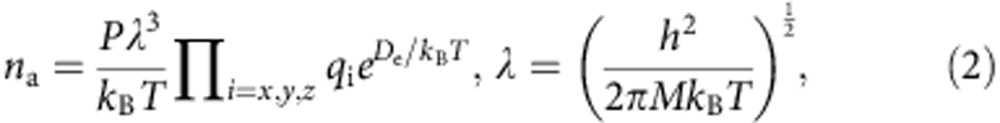


where *P*, λ, *M*, *k*_B_, *T* and *h* stand for the partial pressure, thermal wavelength and mass of the target gas species, the Boltzmann constant, the temperature and the Planck's constant, respectively, and 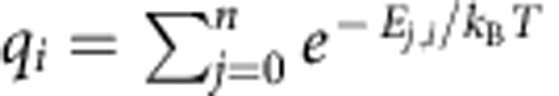 is the one-dimensional partition function. For NO_2_ adsorption on phosphorene, the energy barrier Φ_Ba_ of 0.18 eV for its migration along the armchair direction (the inset of Fig. 3c) suggests that it is localized and cannot be viewed as mobile across the phosphorene surface at room temperature. Instead, the localized gas molecule can be approximated as a harmonic oscillator (reasonable near the potential bottom and only the lower energy levels are of interest, as they are mostly occupied) with the nondegenerate energy level *E*_n_=(*n*+1/2)*hv* and the frequency 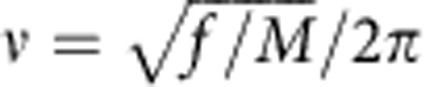, in which *f* is the fitted force constant. Around the bottom of the migration potential, the frequency is found to be 0.22 THz, which is typical for localized gas molecules. Along the zigzag direction, the energy barrier Φ_*Bz*_ of 0.08 eV implies that the gas molecules are quasi-localized around room temperature. Here we treat the gas molecules to be mobile along the zigzag direction and eliminate this approximation-induced overestimation factor that is around exp(2Φ_*Bz*_/*k*_B_*T*) in the partition function. With the NO_2_ concentration at 20 p.p.b. and the temperature at 300 K, equation (2) reveals that the adsorption density of NO_2_ on phosphorene is 2.8 × 10^12^ cm^−2^—this is quite high and superior to the adsorption density of NO_2_ (2.0 × 10^10^ cm^−2^) at 20 p.p.b. concentration on graphene at room temperature in our previous calculations^29^.

We then estimated the adsorption densities of CO, H_2_S and H_2_, to understand the insensitivity of the PNS towards them. Owing to their much smaller binding energies (*E*_b_ is 0.23, 0.14 and 0.055 eV for CO, H_2_S and H_2_, respectively; also see Supplementary Fig. 11 for adsorption sites and molecular orientations), the upper limit for adsorption density is described by^29^


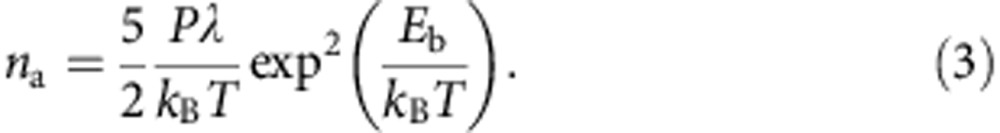


Figure 3d plots the resulting adsorption densities, which are 1.32 × 10^11^, 1.10 × 10^8^ and 0.62 × 10^6^ cm^−2^ for CO, H_2_S and H_2_ at 20 p.p.b. at 300 K, respectively. Evidently, they are several orders of magnitude smaller than that of NO_2_. We can also compare the gas concentrations of CO, H_2_S and H_2_ under the same adsorption level of NO_2_. For example, to achieve the adsorption level of 20 p.p.b. NO_2_, the required concentrations of CO, H_2_S and H_2_ are 0.42 × 10^3^, 0.50 × 10^6^ and 0.90 × 10^8^ p.p.b., respectively (Fig. 3d).

### Gas adsorption-induced change in electronic properties

The adsorption density for each individual gas species is necessary but insufficient to fully understand the distinct behaviours of PNS sensors observed in Fig. 2. For example, the NO_2_ adsorption on PNS exhibits two distinct response modes, yet PNS is still insensitive to CO, H_2_S and H_2_ even at very high concentrations and the sensitivity of PNS is thickness dependent. Thus, we investigated the effect of gas adsorption on the electronic structures of phosphorene.

**Figure 4:**
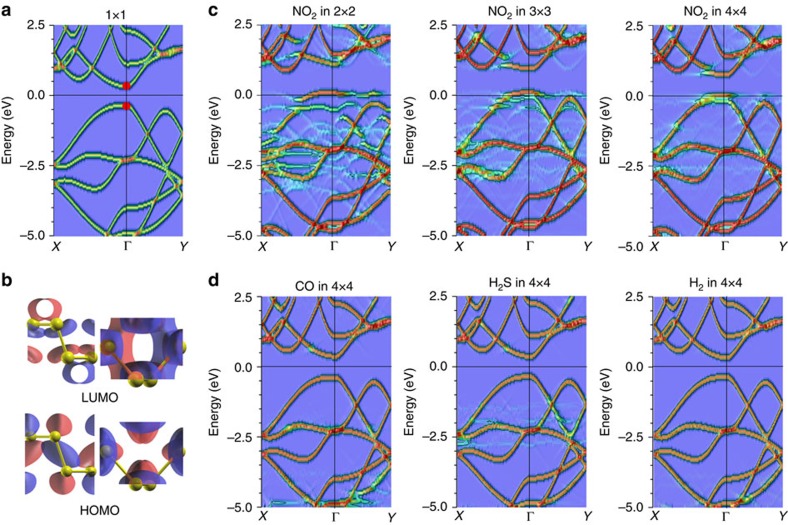
Band structures of phosphorene on gas adsorption. (**a**) The band structure of pristine phosphorene in its primitive cell. (**b**) Side views of charge density distribution contour (0.05 electron per Bohr^3^) of the lowest unoccupied molecular orbital (LUMO) and HOMO states at the Γ point (indicated by the red dots in **a**) of phosphorene. The dummy colours indicate the parities of atomic orbitals. (**c**) From left to right: the band structures projected into the unit cell for the single NO_2_ molecule adsorbed in the 2 × 2, 3 × 3 and 4 × 4 phosphorene super cells, respectively. (**d**) From left to right: the projected band structures of 4 × 4 phosphorene super cells with single CO, H_2_S and H_2_ molecule adsorbed, respectively. The reciprocal paths in **a**,**c** and **d** are selected such that the XΓ and ΓY represent the zigzag (that is, *Y* in Fig. 3a) and armchair (that is, *X* in Fig. 3a) directions in the real space. The electronic structure plot shows the spatial localization of the eigenstates, indicating the relative intensity varying from low (blue) to high (red). The stray states buried in the blue background mainly come from the local region of phosphorene with NO_2_ molecule adsorption. The *k*-projection ‘unfolds' the bands of the supercell from the smaller Brillouin zone to the larger primitive one by decomposing the wave function into different *k* values that can mix in the supercell and essentially provides the straightforward information on the effect of the NO_2_ molecule on the electronic properties of phosphorene on adsorption.

Figure 4a shows that the pristine single-layer phosphorene is a semiconductor with an underestimated direct band gap of 0.92 eV. The lowest unoccupied molecular orbital state and the highest occupied molecular orbital (HOMO) state are located at the Γ point, which is mainly formed by the *p*_z_ orbitals of the phosphorus atoms (Fig. 4b). As the charge transfers from the *p*_z_ orbitals of the phosphorus atoms to the NO_2_ molecule (Fig. 3b), the lowest unoccupied molecular orbital and HOMO states can be significantly affected on NO_2_ adsorption. For low concentrations such as <100 p.p.b., the NO_2_ molecules affect phosphorene considerably by enhancing the hole concentration, while only slightly (or even negligibly) diminishing its carrier mobility, as the lateral distance between the adsorbed individual NO_2_ molecule is quite large (>2.7 nm). Consequently, the conductance of phosphorene is enhanced linearly with a fast rate corresponding with the high-sensitive mode in Fig. 2c. However, this rate is inevitably lowered by increasing NO_2_ concentrations, because the intermolecular distance significantly decreases (for example, 1.2 nm at 500 p.p.b.) and the adsorbed NO_2_ molecules become important scattering centres, thereby leading to a degraded mobility. To illustrate this effect, Fig. 4c presents the *k*-projected band structures of phosphorene with NO_2_ adsorption in several cell sizes (here the spin effect is neglected, as we only focus on the states from the phosphorus atoms and, moreover, these states are not spin polarized at room temperature due to the smaller energy difference of 0.03 eV per system if the spin effect is considered). Obviously, we can see that the adsorbed NO_2_ molecules introduce a local state around the zone centre, which disrupts the HOMO states into segment like (this is even more severe for a smaller cell size), indicating the obstructed conducting channel for holes that leads to a shorter carrier lifetime or mean free path and thus a smaller mobility. In addition, as expected, all the Fermi levels shift towards the top of the valence bands, suggesting the increase of hole concentration due to the charge transfer. Therefore, the decreased hole mobility of phosphorene on exposure to a relatively high concentration of NO_2_ changes the sensing response from high-sensitive mode to low-sensitive mode.

The insensitivity of phosphorene or PNS towards CO, H_2_S and H_2_ could be mainly attributed to two factors. On one hand, in stark contrast to Fig. 4c, all electronic structures in Fig. 4d are barely altered, even using the same cell size, except for some stray states buried in the background by virtue of weak interactions between gas molecules (CO, H_2_S and H_2_) and phosphorene, resulting in negligible changes in conductance. On the other hand, not every individual incoming gas molecule can be attracted to the phosphorene surface due to thermal fluctuations, because the sticking coefficient/probability is proportional to the binding energy. For CO, H_2_S and H_2_ adsorption on phosphorene, the sticking coefficients at 300 K turn out to be 0.92, 0.81 and 0.59, respectively, whereas it increases to 1.0 for NO_2_ adsorption^29^.

### Modelling the dependence of sensitivity on the PNS thickness

**Figure 5:**
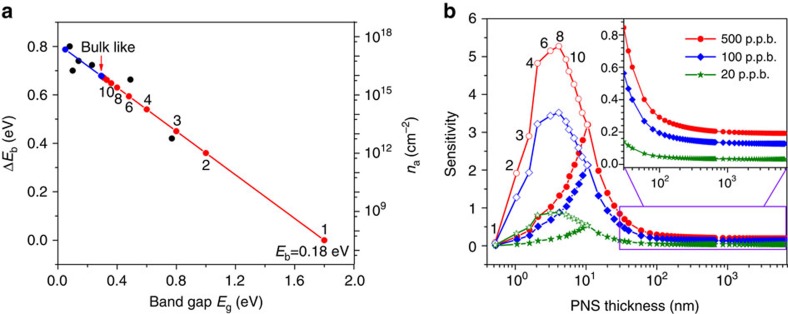
Dependence of sensitivity on the PNS thickness. (**a**) Left axis: binding energy relative to that of the monolayer as a function of band gap. The black dots are the theoretically predicted values with the underestimated band gaps calculated from the conventional DFT calculations, whereas the red dots are the linearly extrapolated ones with respect to the corrected band gaps^13^. The layer number of phosphorene is labelled and the blue dots are the extension of the linear fitting to the region of underestimated band gaps; right axis: the gas molecule adsorption density in 20 p.p.b. NO_2_ concentration at 300 K. (**b**) Sensitivity obtained from equation (5) for 500 p.p.b. NO_2_ and scaled to the concentrations of 100 and 20 p.p.b. with the sensitivity ratio from Fig. 2, respectively. The hollow symbols indicate cases when the effect of mobility degradation is not considered. The inset is the zoomed view of the sensitivity for the PNS thickness from 30 nm to the bulk.

The gas adsorption analysis above is performed on monolayer phosphorene and the experimentally observed sensitivity is dependent on the PNS thickness (*cf*. Fig.2 b,d), even for thick PNS that is bulk like. Thus, it is necessary to take the PNS thickness dependence into account when establishing the model for sensitivity. We first start with the binding energy *E*_b_ variation on different layers of phosphorene. Owing to the severe underestimation of band gaps from the conventional DFT calculation, we fit the calculated binding energies/band gap values and then make the extrapolation with respect to the corrected energy gaps from ref. 13. As shown in Fig. 5a, *E*_b_ increases from the monolayer (0.18 eV) all the way up to and saturates at the bulk limit (0.83 eV), as the PNS with a smaller gap (that is, larger carrier concentration) could enable more charge transfer into the gas molecules. Assuming the energy barriers vary linearly with respect to the binding energy, the corresponding predicted NO_2_ adsorption density by equation (2) is lowest for the monolayer (∼10^7^ cm^−2^) and keeps increasing to the bulk limit (∼10^16^ cm^−2^). For our 4.8-nm-thick sample, the NO_2_ adsorption density is predicted to be ∼10^15^ cm^−2^.

Next, we develop the modelling of sensitivity for semiconductor films (see Supplementary Methods for details) as shown in equation (4)


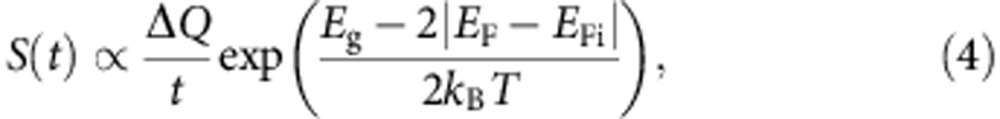


in which 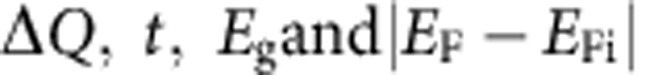 are the total charge transfer from the semiconductor to the gas molecules, the semiconductor thin film thickness, the energy band gap and the energy spacing between the Fermi level and the intrinsic Fermi level, respectively. It is noteworthy that Δ*Q* is also linearly proportional to the gas adsorption density *n*_a_. For the PNS, the energy gap *E*_g_ is dependent on the PNS thickness. With equation (2), we could obtain the sensitivity as


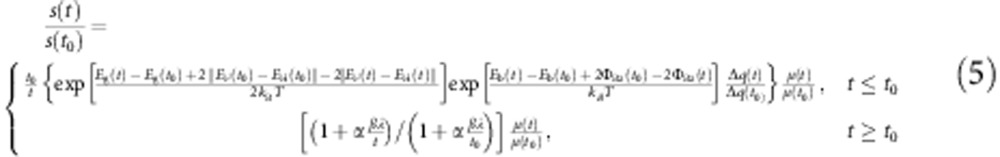


where *t*_0_ (10 nm) is the reference thickness, *λ* (2.9 nm (ref. 13)) is the Thomas–Fermi charge screening length, Δ*q* is the charge transfer into the individual NO_2_ molecule, *μ* is the majority carrier mobility, and *α* and *β* are parameters that need to be obtained from experimental data. Here we assume that Δ*q* is also linearly proportional to *E*_b_ and use the extrapolated Fermi energy spacings^31^. Figure 5b shows the predicted sensitivity with respect to the PNS thickness (from monolayer to bulk) for the NO_2_ concentrations of 500, 100 and 20 p.p.b., respectively. It can be seen that the sensitivity first increases and then decreases as the PNS thickness increases, and the highest sensitivity should be realized experimentally on the PNS thickness ranging from 4.3 to 10+ nm, depending on the PNS quality (that is, the carrier mobility in the PNS). Physically, this dependence arises from the fact that a larger band gap semiconductor has poorer ability to attract the gas molecules due to its lower carrier concentration, whereas the conductivity change is less probable for a smaller band gap semiconductor due to its high carrier concentration. Therefore, there would be an optimum band gap range for the highest sensitivity by balancing these two effects. It is worth noting that the predicted sensitivities for thin PNS could deviate from the experimental values to some extent, owing to the underestimate/overestimate in evaluating the gas adsorption density; however, the predicted trend of sensitivity is reliable.

## Discussion

Our sensitivity model suggests that the highest sensitivity can be realized for the PNS thickness from 4.3 to 10+ nm and the actual optimum value depends on the substrate quality that has an impact on the carrier mobility of PNS. Our experimentally observed optimum thickness of 4.8 nm is just within this range and validates our model. We propose that the dependence of sensitivity on the PNS thickness can be categorized into two regimes as shown by equation (5). For the thinner PNS with a thickness-dependent band gap, the sensitivities are dictated by the band gap. Physically, this dependence arises from the fact that semiconductors with larger/smaller band gaps have poorer/better ability to attract gas molecules because of their lower/higher carrier concentrations (that is, the poorer/better ability for charge transfer to occur between the semiconductor and the gas molecule). As we define the sensitivity as the relative conductance change Δ*G* normalized to the initial conductance *G*_0_ (Δ*G*/*G*_0_), the PNS with a larger/smaller band gap has both a smaller/larger initial conductance *G*_0_ and a smaller/larger conductance change Δ*G* due to the lower/higher gas adsorption density. Although both Δ*G* and *G*_0_ decrease/increase exponentially for the PNS with a larger/smaller band gap, the rates of their changes, namely, their dependences on the band gap, are different due to the different coefficients in the exponential terms (see Supplementary Fig. 13). Consequently, by balancing the different change rates of Δ*G* and *G*_0_, the highest sensitivity would occur at the band gap when the increasing/decreasing rate of Δ*G* begins to decrease/increase as *G*_0_ (that is, the band gap) decreases/increases. Although for the thicker PNS, despite the invariant band gap, the out-of-plane conductivity is much smaller than the in-plane one due to the layered 2D nature of PNS. As a result, the charge transfer on the gas adsorption is not uniformly distributed in the entire sheet compared with the case for the thinner PNS, but is instead accumulated near the top surface region. In our device architecture, the metal electrodes are seated on the top surface of the PNS. On the application of a source-drain current, the carriers are pushed inwards, but with only a limited penetration depth defined as the effective thickness region within which the carrier concentration is larger than the remaining region of the PNS. In the framework of a parallel capacitor model^32^, the PNS conductivity is governed by the effective thickness region after the gas adsorption. Nevertheless, this effective thickness region would still vary with respect to the PNS thickness, as shown in Supplementary Fig. 12, and it increases gradually and finally saturates as the actual PNS thickness increases. Essentially, the effective thickness in the thicker PNS functions similar to the band gap in the thinner PNS in tuning the sensitivity, except that the sensitivity varies monotonically with respect to the effective thickness. As the effective thickness region determines the PNS conductivity, a smaller effective thickness (that is, larger change of PNS conductivity induced by the adsorbed gas molecules) corresponds to a higher sensitivity. Overall, the sensitivity decreases as the effective thickness (actual PNS thickness) increases.

In conclusion, we successfully fabricated and demonstrated high-performance FET sensors based on PNS. Our sensing measurements show that PNS sensors are among the best sensors towards NO_2_ detection at the p.p.b. level in dry air. The sensitivity is dependent on the PNS thickness and 4.8-nm-thick PNS shows the best sensitivity. We also established a statistical thermodynamics model with inputs from first-principles calculations, to characterize the gas adsorption density quantitatively with respect to thickness. Our sensitivity model further suggests that the highest sensitivity can be realized for PNS thicknesses ranging from 4.3 to 10+ nm and the actual optimum value depends on the substrate quality that has an impact on the carrier mobility of PNS. Our experimentally observed optimum thickness of 4.8 nm is within this range and validates our model. In addition, our PNS sensor shows excellent stability in dry air environment and demonstrates high selectivity to NO_2_ gas in the presence of H_2_, CO and H_2_S gases. It is noteworthy that our statistical thermodynamics model can also be applicable to other 2D layered materials, which would advance the understanding of sensing performance and offer insights into the materials selection and design for gas sensor applications. Overall, the superior chemical-sensing performance of PNS-based FET devices can potentially open up exciting opportunities for a wide range of applications, such as environment monitoring and industrial process control at room temperature.

During the revision of this study, we became aware of the work by Zhou *et al*.^33^ that also demonstrated the superior sensing performance of BP with a sensitivity of 20% for 20 p.p.b. NO_2_ in an argon atmosphere.

## Methods

### Sample preparation and characterization

BP crystals were purchased from HQ Graphene and exfoliated by the ‘Scotch tape' method onto degenerately doped <100> Si wafers with a 300-nm-thick thermal oxide. Features were defined with electron-beam lithography in poly(methyl methacrylate). To make electrical contact to the flake, 20 nm of Ni and 40 nm of Au were used as contact metals. After deposition, the PNS served as the conducting channel, bridging the gap of the gold fingers (2 μm). An annealing treatment at 200 °C for 1 h in Ar atmosphere was performed, to improve the contacts between the PNS and gold electrodes. The sensor was further identified and confirmed by a scanning electron microscope (Hitachi S4800) and a Raman spectrometer (Renishaw 1000B). To determine the thickness of the exfoliated BP, AFM was performed using both an Agilent Technology 5420 AFM with a cantilever (Nanosensors PPP-NCH), as well as a Bruker Dimension FastScan with ScanAsyst. The structure of the exfoliated PNS was characterized by TEM (Hitachi H-9000-NAR). High-resolution TEM and selected area electron diffraction (at an acceleration voltage of 300 kV) were used to characterize the crystal structure of the PNS.

### Vacuum charge transport measurements

Electrical characterization of PNS devices were performed with a Lakeshore CRX 4K at a pressure of ∼10^−4^ Torr, using two Keithley Source Meter 2400 (Keithley, Cleveland, OH) to measure electrical characteristics at room temperature.

### Sensing test

The sensing tests were performed in an air-tight chamber with electrical feedthroughs. A constant voltage was applied to the device (between the source and the drain electrodes) and the variation of electrical conductance was monitored and recorded with the changes in the gas environment using a Keithley 2602 Source Meter (Keithley). A typical sensing measurement cycle consisted of three sequential steps: (1) dry air (dew point −62 °C) flow was introduced into the chamber to record a baseline; (2) a target gas (NO_2_) balanced in dry air was injected to register sensing signals; and (3) the sensor was recovered in a dry air flow. All gas flows were precisely controlled using mass-flow controllers.

### DFT calculations

The DFT calculations were performed by the code OPENMX^34^. The electron wavefunctions were expanded by the pseudo atomic orbitals and specified by P7.0-*s*^2^*p*^2^*d*^1^, S7.0-*s*^2^*p*^2^*d*^1^, C5.0-*s*^2^*p*^2^*d*^1^, N5.0-*s*^2^*p*^2^*d*^1^, O5.0-*s*^2^*p*^2^*d*^1^ and H5.0-*s*^2^*p*^2^ for P, S, C, N, O and H, respectively, where 7.0 (5.0) represents the cutoff atomic radius in Bohr and *s*^2^*p*^2^*d*^1^ indicates that the basis functions are expanded by two primitive orbitals of individual *s* and *p* orbitals and one primitive orbital of individual *d* orbital. The core electrons were treated by the norm-conserving pseudopotential with the exchange–correlation functional in the framework of the generalized gradient approximation (GGA) of Perdew–Burke–Ernzerhof^35^ with and without spin polarization for NO_2_ and other gases, respectively. The modified version of the DFT-D method^36^ in the semi-empirical GGA functional was adopted to incorporate the van der Waals force in the gas adsorption systems considered here using the damped atom pairwise dispersion corrections in the form of C_6_·R^−6^, which has been shown to be very successful in describing the medium-to-large range interactions^37^. The numerical integrations were performed with a cutoff energy of 2,720 eV in real space and a *k*-point directional density of 0.02 Å^−1^ in the reciprocal space. The structural relaxations were terminated with the force criteria of 0.01eV Å^−1^. A layer spacing larger than 20 Å was used for the single gas molecule adsorption so that suspicious interactions could be ignored between the adjacent layers and between the molecules themselves due to the periodic boundary conditions. The transition barrier was searched by the nudged elastic band method^38^. The *k*-projected band structures were implemented by *flair*^39^ using the full-potential linearized-augmented plane-wave method with the valence electrons treated by GGA. The 2D experimental lattice constants of BP (*a*=3.314 Å, *b*=4.376 Å and *c*=5.25 Å) were used^17^.

## Additional information

**How to cite this article:** Cui, S. *et al*. Ultrahigh sensitivity and layer-dependent sensing performance of phosphorene-based gas sensors. *Nat. Commun.* 6:8632 doi: 10.1038/ncomms9632 (2015).

## Supplementary Material

Supplementary Information Supplementary Figures 1-13, Supplementary Table 1, Supplementary Notes 1-3, Supplementary Methods and Supplementary References
