# Supplementary material for: Ultrahigh sensitivity and layer-dependent sensing performance of phosphorene-based gas sensors
Source: Nat Commun. 2015 Oct 21;6:8632. doi: 10.1038/ncomms9632 (PMC4639804; doi:10.1038/ncomms9632)
Supplement: Supplementary Information — Supplementary Figures 1-13, Supplementary Table 1, Supplementary Notes 1-3, Supplementary Methods and Supplementary References [file ncomms9632-s1.pdf]

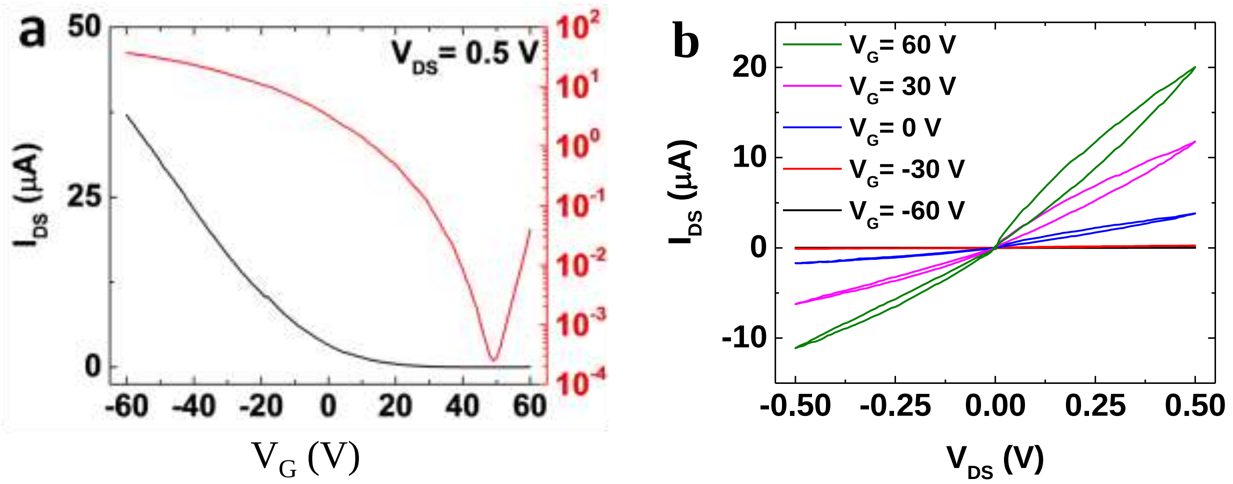

Supplementary Figure 1. (**a**) FET curve and (**b**) I-V curve of the 4.8 nm-thick PNS device in vacuum. The drain-source voltage was 0.5 V for the FET transfer curve measurement. In the output measurement, the drain-source voltage starts at 0 V and goes to 0.5V, then goes to -0.5 V and finally back to 0 V.

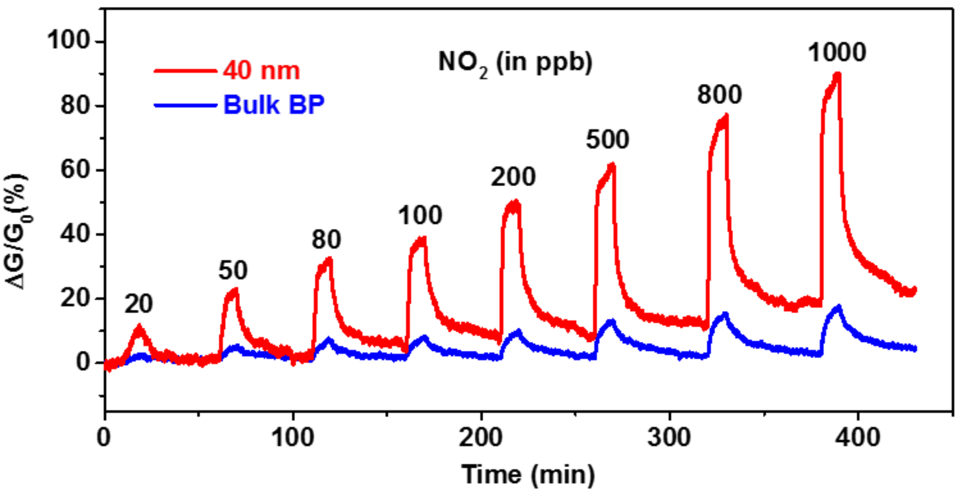

Supplementary Figure 2. Dynamic response curves of relative conductance change versus time for NO_2 concentrations ranging from 20−1,000 ppb for 40-nm-thick and bulk BP sensors.

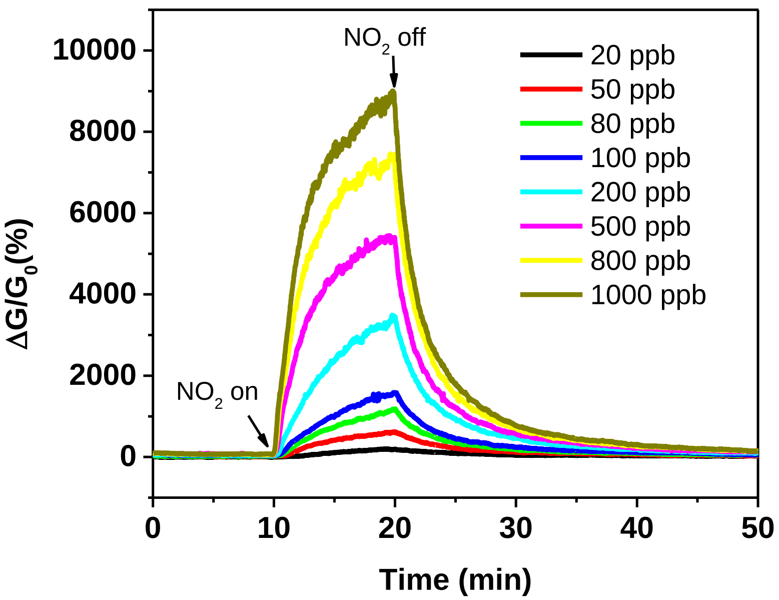

Supplementary Figure 3. Overlapping response curves of 4.8-nm-thick PNS sensor using the same data in Fig. 2a.

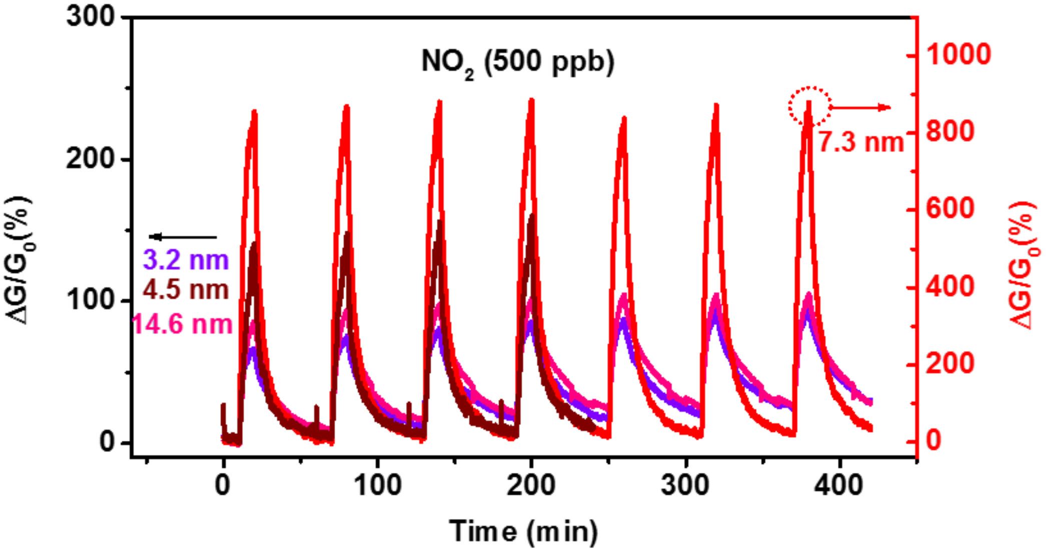

Supplementary Figure 4. Sensing response of PNS upon exposure to 500 ppb NO_2 in air.

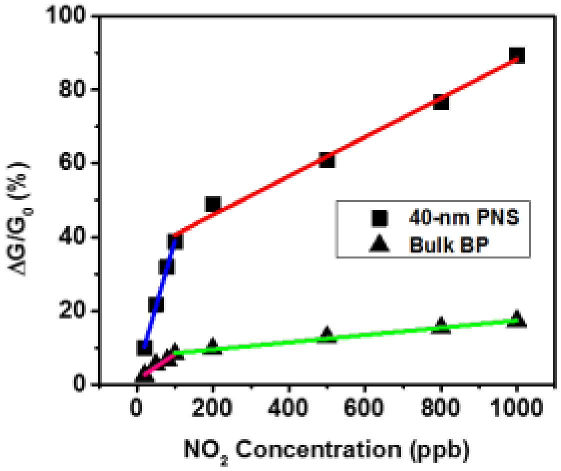

Supplementary Figure 5. Calibration curves of a 40 nm-thick PNS sensor and a bulk BP sensor to NO_2 gas.

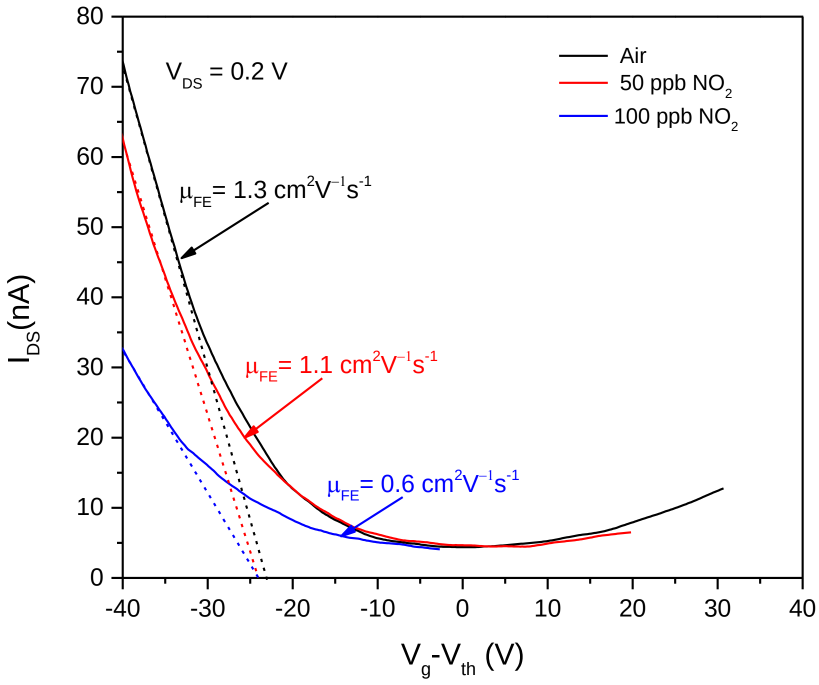

Supplementary Figure 6. FET characteristics of the 6-nm PNS sensor before and after 10-min exposure against NO_2 in concentrations of 50 and 100 ppb in dry air.

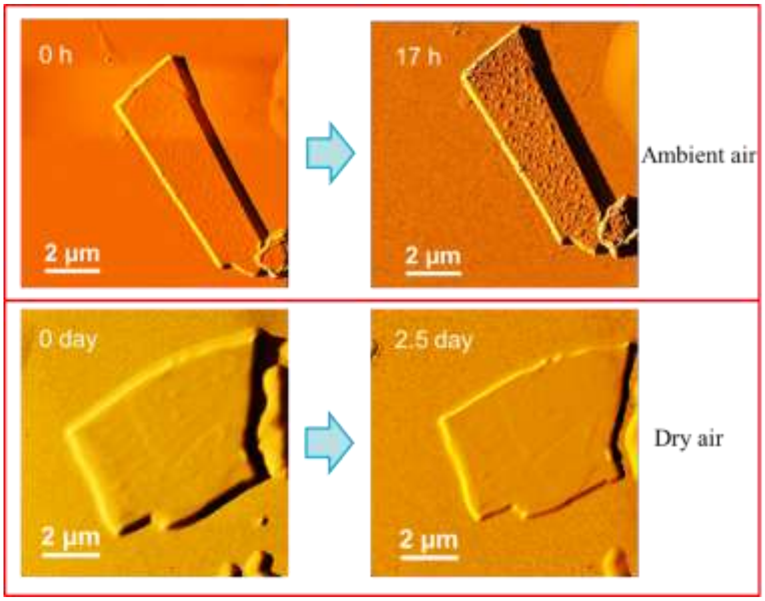

Supplementary Figure 7. AFM images of PNS aging in ambient air and dry air.

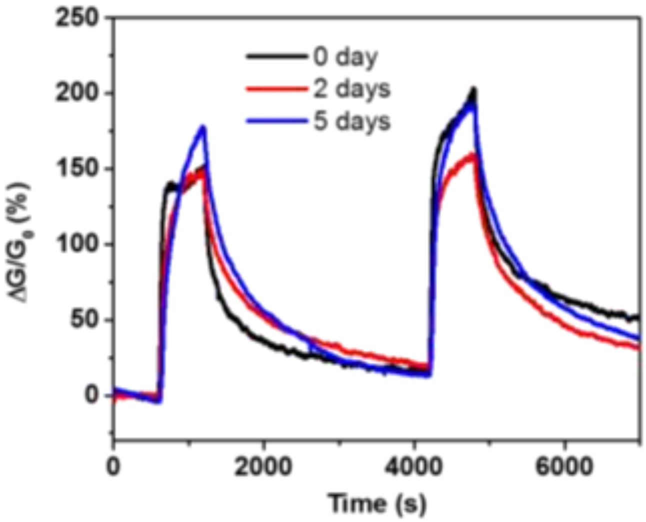

Supplementary Figure 8. Dynamic sensing response of 40-nm-thick PNS to 500 ppb NO_2 in dry air.

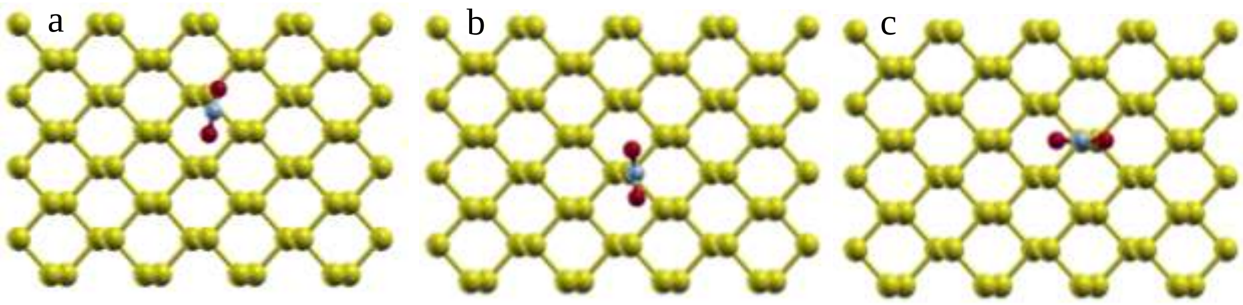

Supplementary Figure 9. Top view of different adsorption sites of NO_2 with a relative binding energy of 0 eV, -0.11 eV, and -0.14 eV for (a), (b), and (c), respectively.

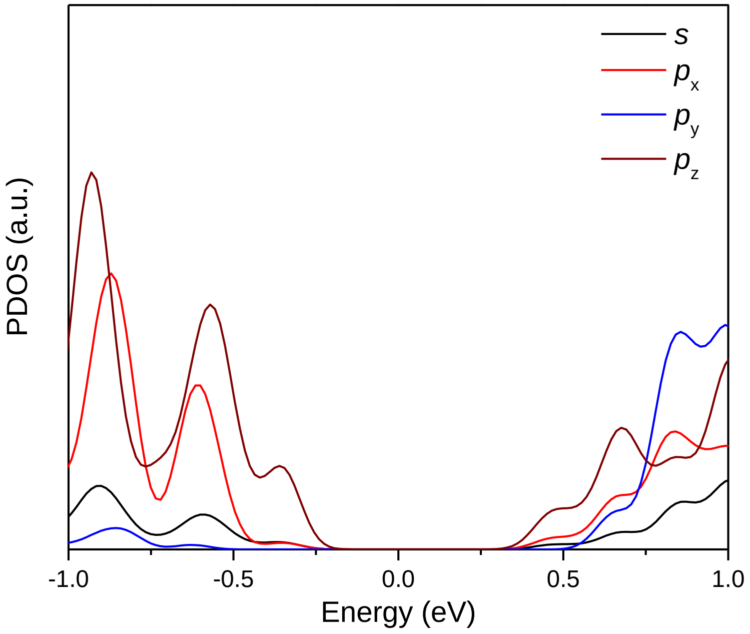

Supplementary Figure 10. Partial density of states (PDOS) of pristine monolayer phosphorene resolved to each atomic orbital of the valence electrons. The states are calculated using the Gaussian method with broadening of 0.075 eV.

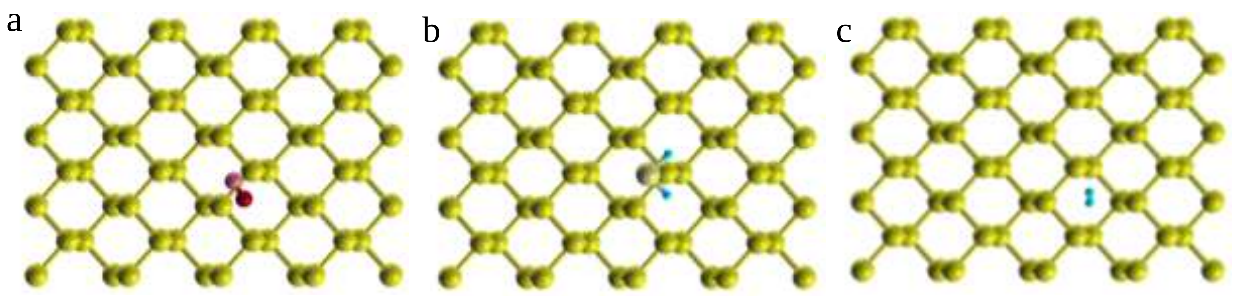

Supplementary Figure 11. Energetically favored adsorption models of (**a**) CO, (**b**) H_2S, and (**c**) H_2.

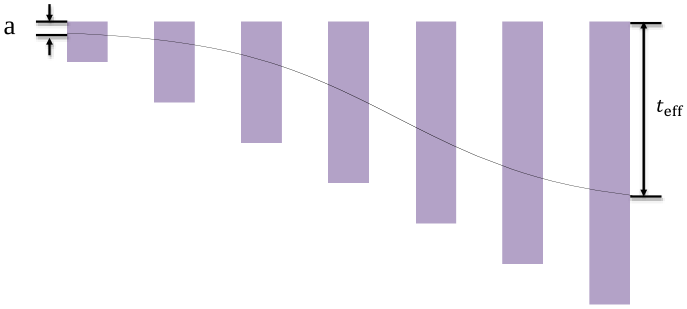

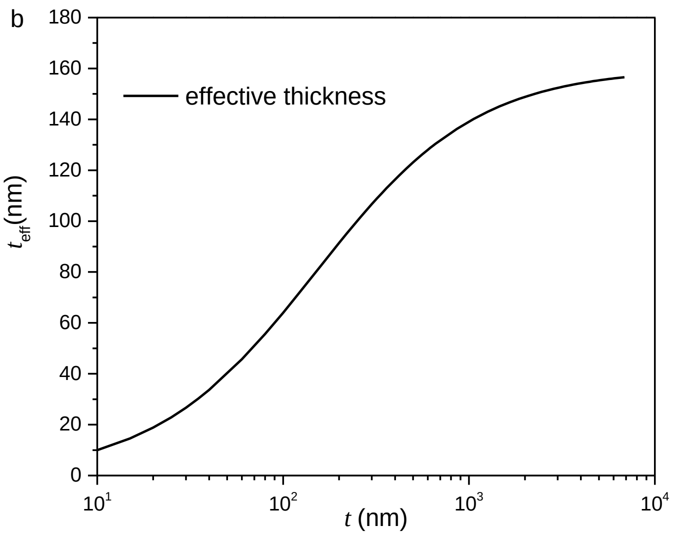

Supplementary Figure 12. **a** Schematic of the effective thickness variation with respect to the actual PNS thickness. The colored bar indicate the actual PNS thickness t. **b** The predicted effective PNS thickness using the supplementary Eq. (15).

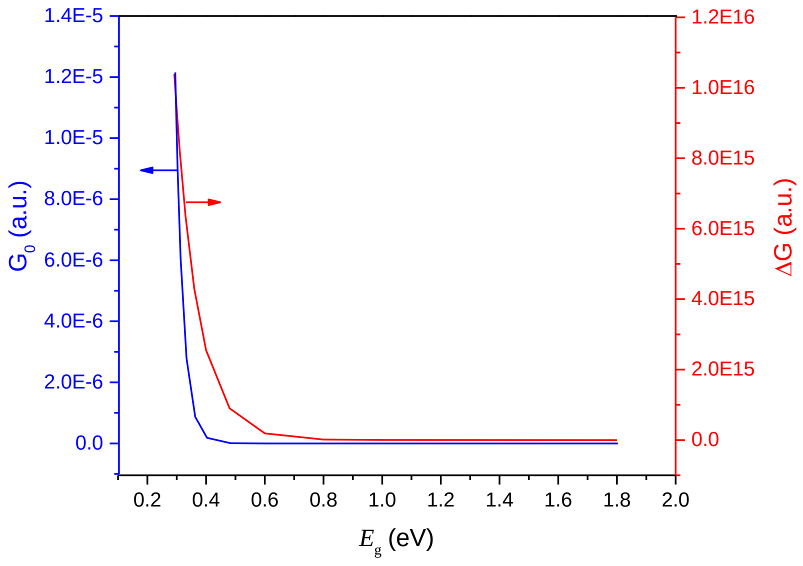

Supplementary Figure 13. The initial conductance G_0 (left axis) and the relative conductance change ΔG (right axis) as a function of energy band gap of the PNS.

Supplementary Table 1 | Detection limits and corresponding sensitivities ($\Delta G/G_0$) of various sensing materials towards NO_2.

	Va-RGO[4]	RGO/Cu_2O[5]	CMG[6]	S-SWCNT[7]	In_2O_3[8]	MoS_2/PtNPs[9]	Our work
Lower detection limit (ppb)	50	64*	70*	44*	5	2*	20
$\Delta G/G_0$ (%)	10	3*	11*	0.15*	-20	0.025*	190

* indicates that the lower detection limit and sensitivity are extrapolated from the calibration curve.

Supplementary Note 1

To quantify the mobility degradation, we extracted the field-effect mobilities from the FET curves of a 6-nm PNS sensor before and after 10-min exposure against NO_2 in concentrations of 50 and 100 ppb (Supplementary Fig. 6). Clearly, the field-effect carrier mobility in air 1.3 $cm^2V^{-1}s^{-1}$ only slightly degraded to 1.1 $cm^2V^{-1}s^{-1}$ upon exposure to 50 ppb NO_2. However, it greatly reduced to 0.6 $cm^2V^{-1}s^{-1}$ when the gas concentration increased to 100 ppb NO_2, implying the transition from the high-sensitive mode to the low-sensitive mode. Note that the critical gas concentration for the transition from the high-sensitive mode to the low-sensitive mode is dependent on the PNS thickness. For example, this critical gas concentration for the 6 nm PNS sensor is very similar to those of thicker films (cf. supplementary Fig. 5 for the 40 nm PNS and bulk BP) and is lower than that (~200 ppb) of the 4.8 nm PNS sensor. This is because the band gap difference (~ 0.2 eV) between 6 nm and 40 nm PNS is smaller than that (~ 0.4 eV) between 4.8 nm and 6 nm PNS[7]. Therefore, the critical gas concentration for the mode transition for the 6 nm PNS sensor is closer to that for the 40 nm PNS than that for the 4.8 nm PNS (as also shown in Fig. 5a that films with a smaller band gap will have a larger gas adsorption density upon exposure to the same gas concentration).

Note that the carrier mobilities in the supplementary Fig. 6 are quite small, which can be ascribed to several factors. Firstly, the impurities trapped between the substrate and PNS would scatter the transport of carriers and lead to a decrease in the carrier lifetime and thus carrier mobility. The carrier mobility of our 6 nm-thick PNS sample in vacuum was measured as 61 $cm^2V^{-1}s^{-1}$, similar to the reported value of 55 $cm^2V^{-1}s^{-1}$ for a 5 nm-thick BP flake[1]. Secondly, upon exposure to air,

the surface adsorbed gas molecules further degrade the carrier mobility through scattering. This is also evidenced in Castellanos-Gomez *et al.*'s work[2] that showed the on-state current of few-layer BP flakes dropped by one to two orders of magnitude even right after breaking the vacuum with no surface oxidation occurring. Finally, BP is an anisotropic material and the carrier mobility varies along different directions with the maximum mobility along the armchair direction[3]. However, the orientation of metal electrodes on the PNS surface is random and varies from zigzag to armchair.

Supplementary Note 2

For DFT calculations, we started the gas adsorption with various adsorption sites (atop site: top of phosphorus atom; bridge site: middle of the bond between two neighboring phosphorus atoms; and hollow site: the center of the phosphorus ring) and different molecular orientations (the oxygen atoms in NO_2 either pointing upwards or downwards), and then relax the systems. The energetically favored NO_2 adsorption on phosphorene turns out to be the bridge site, with the N atom sitting above the middle of the phosphorus bond and the two O atoms pointing towards the two P atoms underneath, as shown in Fig. 3a. The binding energy E_b of NO_2 is defined as $E_b= E_{gas}+E_P - E_{gas+P}$ where E_{gas}, E_P and E_{gas+P} are the energies of a single gas molecule, pure phosphorene, and the complex system, respectively.

Supplementary Note 3

The phosphorus atoms form the sp^3-like bonding, but with one dangling bond that could interact weakly with its nearest neighbors due to the puckered honeycomb structure. In contrast to the

sp^2 bonding in graphene, which has only one extra electron in the p_z orbital that forms the π bond responsible for NO_2 adsorption, electrons of the primitive s and p_x, p_y, and p_z orbitals in the one dangling sp^3 orbital equivalent to one electron are also available for attracting NO_2 molecules. In other words, there are about two extra effective electrons in each phosphorus atom compared with one effective electron in each aromatic carbon atom that interacts with the NO_2 molecule. This can also be seen by the atomic orbital-resolved density of states in Supplementary Fig. 10, in which s, p_x, p_y, and p_z orbitals show the similar shape (i.e., suggesting the orbital hybridization), but the p_z orbital is mostly populated by the charge associated with bands close to the Fermi level.

Supplementary methods

In semiconductors, the electron and hole concentrations are

$$n_{\mathrm{e}} = 2\left(\frac{m_{\mathrm{e}}k_{\mathrm{B}}T}{2\pi\hbar^2}\right)^{\frac{3}{2}} exp\left(\frac{E_{\mathrm{F}}-E_{\mathrm{C}}}{k_{\mathrm{B}}T}\right), n_{\mathrm{h}} = 2\left(\frac{m_{\mathrm{h}}k_{\mathrm{B}}T}{2\pi\hbar^2}\right)^{\frac{3}{2}} exp\left(\frac{E_{\mathrm{V}}-E_{\mathrm{F}}}{k_{\mathrm{B}}T}\right), \quad (1)$$

in which $m_{\mathrm{e}}(m_{\mathrm{h}})$ is the electron (hole) mass, k_{B} is the Boltzmann constant, T is the temperature, $\hbar$ is the Planck's constant, E_{F} is the Fermi level, E_{C} (E_{V}) is the conduction (valence) band edge energy. From the supplementary Eq. (1), we could obtain the following expressions straightforwardly,

$$n_{\mathrm{e}}n_h = 4(\frac{k_{\mathrm{B}}T}{2\pi\hbar^2})^3 \, (m_{\mathrm{e}}m_{\mathrm{h}})^{\frac{3}{2}} exp\left(-\frac{E_{\mathrm{g}}}{k_{\mathrm{B}}T}\right) \quad (2)$$

and

$$\frac{n_{\mathrm{e}}}{n_{\mathrm{h}}} = (\frac{m_{\mathrm{e}}}{m_{\mathrm{h}}})^{\frac{3}{2}} \exp[\frac{2E_{\mathrm{F}}-(E_{\mathrm{C}}+E_{\mathrm{V}})}{k_{\mathrm{B}}T}] = (\frac{m_{\mathrm{e}}}{m_{\mathrm{h}}})^{\frac{3}{2}} exp\left[\frac{2(E_{\mathrm{F}}-E_{\mathrm{Fi}})}{k_{\mathrm{B}}T}\right] \approx exp\left[\frac{2(E_{\mathrm{F}}-E_{\mathrm{Fi}})}{k_{\mathrm{B}}T}\right]. \quad (3)$$

Here, $E_g = E_C - E_V$ is the band gap, and E_{Fi} is the intrinsic Fermi level.

The conductivity of a semiconductor is

$$\sigma = e(n_e\mu_e + n_h\mu_h) \tag{4}$$

where e is the elementary charge and $\mu_e(\mu_h)$ is the electron (hole) mobility. We define the conductivity ratio from the electron and hole part as,

$$R = \frac{en_e\mu_e}{en_h\mu_h} \; or \; \frac{en_h\mu_h}{en_e\mu_e} \approx \frac{n_e}{n_h} \; or \; \frac{n_h}{n_e} = exp\left[\frac{2|E_F - E_{Fi}|}{k_B T}\right] \geq 1 \tag{5}$$

for n-type or p-type semiconductors. Note that in the supplementary Eq. (5), we assume $\mu_e \approx \mu_h$ for simplicity and the difference between them is mostly less than one order of magnitude. For specific materials, this difference can be considered for better accuracy. After the following algebra,

$$R = \frac{n_e}{n_h} \rightarrow n_h = \frac{n_e}{R} \text{ and thus } n_e n_h = n_e\left(\frac{n_e}{R}\right) = \frac{n_e^2}{R} \rightarrow n_e = (n_e n_h R)^{\frac{1}{2}},$$

we obtain

$$n_e = 2\left(\frac{k_B T}{2\pi\hbar^2}\right)^{\frac{3}{2}} (m_e m_h)^{\frac{3}{4}} \left\{exp\left[-\frac{E_g - 2(E_{Fn} - E_{Fi})}{k_B T}\right]\right\}^{\frac{1}{2}} \tag{6}$$

and

$$n_h = 2\left(\frac{k_B T}{2\pi\hbar^2}\right)^{\frac{3}{2}} (m_e m_h)^{\frac{3}{4}} \left\{exp\left[-\frac{E_g - 2(E_{Fi} - E_{Fh})}{k_B T}\right]\right\}^{\frac{1}{2}} \tag{7}$$

for the n-type and p-type semiconductors with Fermi level $E_{Fn}$and E_{Fh} , respectively.

In conductance based gas sensors, the sensitivity for n- or p-type semiconductors is defined as

$$S = \frac{\sigma - \sigma_0}{\sigma_0} = \frac{e(n_\mathrm{e}\mu_\mathrm{e} - n_\mathrm{e0}\mu_\mathrm{e0})}{e(n_\mathrm{e0}\mu_\mathrm{e0} + n_\mathrm{h0}\mu_\mathrm{h0})} \; or \; \frac{e(n_\mathrm{h}\mu_\mathrm{h} - n_\mathrm{h0}\mu_\mathrm{h0})}{e(n_\mathrm{e0}\mu_\mathrm{e0} + n_\mathrm{h0}\mu_\mathrm{h0})} \tag{8}$$

where σ_0 and σ are the conductance before and after the target gas adsorption. For low gas concentrations, the gas adsorption density is small and the gas distribution is so sparse that the gas adsorption induced scattering effect on the carrier mobility can be neglected (i.e., $\mu_\mathrm{e} \approx \mu_\mathrm{e0}$ and $\mu_\mathrm{h} \approx \mu_\mathrm{h0}$). Therefore, the supplementary Eq. (8) is then reduced to

$$S = \frac{\Delta n_\mathrm{e}}{n_\mathrm{e0} + n_\mathrm{h0}} \; or \; \frac{\Delta n_h}{n_\mathrm{e0} + n_\mathrm{h0}} \tag{9}$$

For n-type or p-type semiconductors, $n_\mathrm{e0} \gg n_\mathrm{h0}$ or $n_\mathrm{h0} \gg n_\mathrm{e0}$ holds and thus,

$$\begin{aligned} S &= \frac{\Delta n_\mathrm{e}}{n_\mathrm{e0}} \; or \; \frac{\Delta n_\mathrm{h}}{n_\mathrm{h0}} \\ &= \frac{\Delta Q/t}{n_\mathrm{e0}} \; or \; \frac{\Delta Q/t}{n_\mathrm{h0}} \\ &= \frac{\left\{\frac{\Delta Q}{t}\left[exp\left(\frac{E_\mathrm{g} - 2(E_\mathrm{Fn} - E_\mathrm{Fi})}{k_\mathrm{B}T}\right)\right]^{\frac{1}{2}}\right\}}{2\left(\frac{k_\mathrm{B}T}{2\pi\hbar^2}\right)^{\frac{3}{2}}(m_\mathrm{e}m_\mathrm{h})^{\frac{3}{4}}} \; or \; \frac{\left\{\frac{\Delta Q}{t}\left[exp\left(\frac{E_\mathrm{g} - 2(E_\mathrm{Fi} - E_\mathrm{Fp})}{k_\mathrm{B}T}\right)\right]^{\frac{1}{2}}\right\}}{2\left(\frac{k_\mathrm{B}T}{2\pi\hbar^2}\right)^{\frac{3}{2}}(m_\mathrm{e}m_\mathrm{h})^{\frac{3}{4}}}. \end{aligned} \tag{10}$$

In a compact form, the supplementary Eq. (10) can be reformulated as

$$\mathrm{S} = \frac{\left\{\frac{\Delta Q}{t}\left[exp\left(\frac{E_\mathrm{g} - 2|E_\mathrm{F} - E_\mathrm{Fi}|}{k_\mathrm{B}T}\right)\right]^{\frac{1}{2}}\right\}}{2\left(\frac{k_\mathrm{B}T}{2\pi\hbar^2}\right)^{\frac{3}{2}}(m_\mathrm{e}m_\mathrm{h})^{\frac{3}{4}}}. \tag{11}$$

Here, ΔQ is charge transfer per planar unit area in the 2D film that can be determined with the knowledge of gas adsorption density (cf. Eqs. (2) and (3) in the main text) and t is the film thickness.

Since the sensitivity is dependent on the PNS thickness, the sensitivity ratio can be formulated as

$$\frac{s(t)}{s(t_0)} = \frac{\mu(t)\left\{\frac{\Delta Q(t)}{t}\left[exp\left(\frac{E_g(t)-2|E_F(t)-E_{Fi}(t)|}{k_B T}\right)\right]^{\frac{1}{2}}\right\}}{\mu(t_0)\left\{\frac{\Delta Q(t_0)}{t_0}\left[exp\left(\frac{E_g(t_0)-2|E_F(t_0)-E_{Fi}(t_0)|}{k_B T}\right)\right]^{\frac{1}{2}}\right\}}$$

$$=\frac{t_0}{t}\frac{n_a(t)\Delta q(t)}{n_a(t_0)\Delta q(t_0)}\frac{\mu(t)}{\mu(t_0)}exp\left[\frac{E_g(t)-E_g(t_0)+2|E_F(t_0)-E_{Fi}(t_0)|-2|E_F(t)-E_{Fi}(t)|}{2k_B T}\right]. \quad (12)$$

Here Δq is the charge transfer of the individual gas molecules. As the band gap of PNS decreases when the PNS thickness increases (especially from monolayer up to ~10 nm, then barely changes and keeps constant at the bulk value), we divide the PNS into the thin (0.5-10 nm) and thick (>10 nm) regions. In the thin region, the carrier mobility is also thickness dependent due to the scattering effect from the substrate impurities. Eq. (2) in the main text is adopted to evaluate the gas adsorption density, and therefore

$$\frac{s(t)}{s(t_0)} = \frac{t_0}{t}\left\{exp\left[\frac{E_g(t)-E_g(t_0)+2|E_F(t_0)-E_{Fi}(t_0)|-2|E_F(t)-E_{Fi}(t)|}{2k_B T}\right]exp\left[\frac{E_b(t)-E_b(t_0)+2\Phi_{Bz}(t_0)-2\Phi_{Bz}(t)}{k_B T}\right]\frac{\Delta q(t)}{\Delta q(t_0)}\right\}\frac{\mu(t)}{\mu(t_0)}. \quad (13)$$

For the thick PNS, as the band gap barely changes, which also holds for the binding energy (and consequently for the charge transfer and adsorption density), the supplementary Eq. (13) is reduced to

$$\frac{s(t)}{s(t_0)} = \frac{t_0}{t}\frac{\mu(t)}{\mu(t_0)}. \quad (14)$$

Note that the thickness t in the supplementary Eq. (14) should be replaced by the effective thickness, because the charge transfer upon the gas adsorption is not uniformly distributed in the entire film due to the layered 2D nature (the out-of-plane conductivity is much smaller than the in-plane one), it is instead accumulated at the surface region with certain penetration depth that would vary with respect to the PNS thickness t as illustrated in the supplementary Fig. 12. Here, we empirically take this effective thickness as

$$t_{\text{eff}} = \frac{(1+\alpha)\beta\lambda}{1+\alpha\frac{\beta\lambda}{t}},\ t \geq \beta\lambda \qquad (15)$$

where λ is the Thomas-Fermi charge screening length, α and β are fitting parameters.

Then the sensitivity for thick PNS is

$$\frac{s(t)}{s(t_0)} = \frac{1+\alpha\frac{\beta\lambda}{t}}{1+\alpha\frac{\beta\lambda}{t_0}}\frac{\mu(t)}{\mu(t_0)}. \qquad (16)$$

To adopt the supplementary Eq. (16) in evaluating the sensitivity, we have to judiciously select the values of α and β that have distinctive physical meanings. As the penetration depth $\beta\lambda$ is equal to the actual PNS thickness t for thin PNS, and then begins to increase but smaller than t. Thus, there exists a critical thickness t_{c}. We choose $t_{\text{c}} = \beta\lambda = 10\ nm$. On one hand, the gas adsorption induced carrier concentration at t_{c} with respect to the surface one is reduced to $exp(-\beta\lambda/\lambda) = 3.2\%$ and thus it holds that the adsorbed gas molecules would affect the carrier concentration within the entire PNS with $t < t_{\text{c}}$; on the other hand, carrier mobility extracted from the field effect measurements shows that the mobility increases from the monolayer to thin PNS with $t = 10\ nm$ and then slightly decreases as the PNS thickness t increases[1]. The physical meaning of α is that t_{eff} will increase with decreasing rate upon the application of source-drain current which pushes the carrier to penetrate through PNS. α (=5) can be obtained by the ratio of sensitivity experimentally measured and ratio of the mobility at different PNS thicknesses.

Supplementary References

1 Li, L., *et al.* Black phosphorus field-effect transistors. *Nat. Nanotechnol.* **9,** 372-377 (2014).

2 Island *et al*, Environmental instability of few-layer black phosphorus. 2D Mater. **2**, 011002 (2015).

3 Qiao *et al*, High-mobility transport anisotropy and linear dichroism in few-layer black phosphorus. *Nat Commun.* **5**, 4475 (2014).

4 Cui, S. *et al.* Ultrasensitive chemical sensing through facile tuning defects and functional groups in reduced graphene oxide. *Anal. Chem.* **86**, 7516-7522 (2014).

5 Deng, S. *et al.* Reduced graphene oxide conjugated Cu_2O nanowire mesocrystals for high-performance NO_2 gas sensor. *J. Am. Chem. Soc.* **134**, 4905-4917 (2012).

6 Yuan, W. J., Liu, A. R., Huang, L., Li, C. & Shi, G. Q. High-performance NO_2 sensors based on chemically modified graphene. *Adv. Mater.* **25**, 766-771 (2013).

7 Li, J. *et al.* Carbon nanotube sensors for gas and organic vapor detection. *Nano Lett.* **3**, 929-933 (2003).

8 Zhang, D. *et al.* Detection of NO_2 down to ppb levels using individual and multiple In_2O_3 nanowire devices. *Nano Lett.* **4**, 1919-1924 (2004).

9 He, Q. Y. *et al.* Fabrication of flexible MoS_2 thin-tilm transistor arrays for practical gas-sensing applications. *Small* **8**, 2994-2999 (2012).
